# Real-life study of the use of anti-VEGF therapy versus dexamethasone implant for treatment of macular edema in retinal vein occlusion

**DOI:** 10.1007/s00417-021-05146-8

**Published:** 2021-03-18

**Authors:** Manuel Casselholm de Salles, David Epstein

**Affiliations:** Karolinska Institutet, St. Erik Eye Hospital, Eugeniavägen 12, 17164 Stockholm, Sweden

**Keywords:** Anti-VEGF, Dexamethasone, Retinal vein occlusion, Macular edema, Real-life

## Abstract

**Purpose:**

To investigate the long-time outcome of patients with branch vein occlusion (BRVO) and central vein occlusion (CRVO) treated with anti-VEGF injections compared to the dexamethasone (DEX) implant.

**Methods:**

This retrospective real-life study included all 492 patients presenting with retinal vein occlusion (RVO) during 2012–2013 at St. Erik Eye Hospital. Maximum follow-up was 5 years.

**Results:**

The mean time of follow-up for patients treated for macular edema was 33.2±17.7 and 34.3±18.1 months in the BRVO and CRVO groups, respectively. At the end of follow-up, the best-corrected visual acuity improved +9.8±20.4 Early Treatment Diabetic Retinopathy Study letters in BRVO patients receiving anti-VEGF therapy while patients treated with the DEX implant lost −2.1±23.4 letters (*p*<0.05). CRVO patients treated with anti-VEGF therapy improved +0.2±27.6 letters while patients receiving a DEX implant lost −9.7±32.6 letters (*p*=0.11). Overall, in RVO patients treated with anti-VEGF injections, the central retinal thickness decreased to 322±174μm compared to 398±174 μm in patients treated with the DEX implant (*p*<0.05).

**Conclusions:**

In a clinical setting, a substantial part of patients is still in follow-up a long time after presentation. The visual and anatomical outcomes were better in patients treated with anti-VEGF agents compared to subjects receiving a DEX implant.
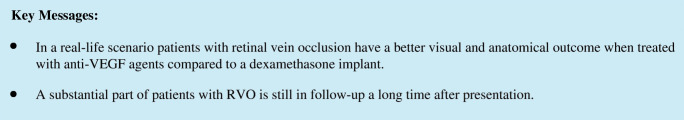

## Introduction

Retinal vein occlusion (RVO) is a common retinal vasculopathy that may cause significant visual impairment [1, 2]. Macular edema (ME) is a major cause of the affected visual function. The introduction of intravitreal therapy has offered an important treatment modality and has improved outcomes in RVO patients presenting with ME [3–7]. In the first prospective pivotal trials, visual gains of +13.9–18.3 Early Treatment Diabetic Retinopathy Study (ETDRS) letters were achieved after 52 weeks while receiving between 8 and 9.8 anti-vascular endothelial growth factor (VEGF) injections [3–7]. The long-term outcome of this patient group is less certain. The HORIZON study aimed to follow prospectively for 24 months patients with ME after RVO who had completed the 12 months BRAVO and CRUISE studies [8]. The mean follow-up was 14 months with only 63% of the patients having a 24-month study visit. At this time point, the best-corrected visual acuity (BCVA) decreased by −0.7 (BRVO) and −4.1 ETDRS letters (CRVO). During this second year of follow-up, the patients received between 2.0 (BRVO) and 3.8 (CRVO) additional ranibizumab injections. The RETAIN study investigated prospectively the outcome of 66 RVO patients after 4 years. They showed a BCVA improvement of +20 and +14 letters for BRVO and CRVO patients, respectively, at the end of follow-up while receiving 14.8 ranibizumab injections (BRVO) and 19.2 injections (CRVO) during this time span [9]. How this data translates into a daily clinical setting with a non-selected patient population is not clear. Several retrospective studies have shown conflicting results. In a small retrospective study, 54 RVO patients with ME were treated with ranibizumab and followed for at least 3 years. At the end of the follow-up, patients with BRVO improved +15.1 letters while patients with CRVO gained +6.9 letters [10]. In the BERVOLT study including 152 eyes treated with bevacizumab, the BCVA increased by 0.25 Log MAR in the BRVO group and decreased by −0.118 Log MAR in the CRVO group after 2 years [11].

Treatment with a sustained-release dexamethasone (DEX) implant (Ozurdex®; Allergan Inc., Irvine, CA, USA) is another important therapy option in patients with RVO [12]. The GENEVA study showed a peak increase in visual outcomes after 2 months with a BCVA gain of 8–10 ETDRS letters. However, this effect was not sustained over time and was not different from sham injections after 6 months [12]. There are sparse data on the real-life long-time outcome of RVO patients treated with the DEX implant. Korobelnik et al showed a one-line gain after 2 years in 375 RVO patients while a 24-month retrospective, real-world study, including 155 eyes did not find any significant change in BCVA at the end of follow-up[13, 14].

The purpose of this study is to evaluate the long-term anatomical and functional outcomes in a large patient cohort and to compare the outcomes of patients treated with anti-VEGF agents and the DEX implant.

## Methods

The study adhered to the tenets of the Declaration of Helsinki and the regional ethical review board in Stockholm approved the protocol.

### Study population

In this retrospective study, all patients presenting at St. Erik Eye Hospital, Stockholm, Sweden, from January 2012 to December 2013 with ICD-10 code H34.8a (CRVO) and H34.8b (BRVO) were included in the study. Only treatment-naive patients were eligible to participate. At baseline, a fundus photography was done to confirm the diagnosis.

### Study design

Data collected included patient demographics (age, sex), concomitant diseases (glaucoma/ocular hypertension, arterial hypertension, and diabetes), best-corrected visual acuity (BCVA), central retinal thickness (CRT), treatment exposure (intravitreal injections), and type of neovascular event (NVG, NVI, NVA, NVD, and NVE). The study population was followed for a maximum of 5 years. All patients had a full ophthalmic examination at each visit. The BCVA was assessed at every study visit using an ETDRS chart at a starting distance of 4 meters. Optical coherence tomography (OCT) was performed at every study visit using Cirrus OCT (Carl Zeiss, Meditec, Dublin, CA). Patients with RVO and ME (CRT > 320 μm) were eligible to receive intravitreal injections. In our clinic, we use either anti-VEGF injections (ranibizumab or aflibercept) or sustained-release DEX as first-line therapy in RVO patients. Patients received either 3 initial consecutive monthly injections of ranibizumab or treatment with a DEX implant. From month 3 to the end of follow-up, patients received reinjections pro re nata (PRN) based on functional and anatomic response parameters. If the ME did not respond to one drug switched therapy could be considered. An analysis comparing patients treated with anti-VEGF injections and the DEX implant was performed. For patients who switched therapy, only data from visits before the change of therapy was included. Follow-up was terminated if there was no sign of disease activity or if vision was low without improvement despite intravitreal therapy.

### Statistical analysis

All efficacy analyses were performed with missing values imputed by last observation carried forward (LOCF) method. For statistical analyses, the independent Student’s *t*-test was used for continuous variables and the Fisher’s exact test (to compare differences in distributions between the groups) were used for categorical data. For continuous variables, mean ± SD was used and counts with percentages for categorical variables. A multiple regression analysis was used to evaluate the association between the baseline characteristics and the visual outcome.

## Results

During the study period, 492 eligible treatment-naïve patients were included, out of which 249 presented with BRVO and 243 with CRVO. Patient demographics and baseline ocular characteristics are presented in Table 1. There were several significant differences between the groups at baseline. Patients with BRVO were significantly younger and had better BCVA, less ME ,and fewer comorbidities (glaucoma/OHT, arterial hypertension) compared to the subjects with CRVO (*p*<0.05). Baseline characteristics of patients treated with anti-VEGF agents and the DEX implant are found in Tables 2 and 3.

**Table 1:** Patient demographics and baseline ocular characteristics

Parameter	BRVO *n*=249	CRVO *n*=243	*p* value
Age (yrs±SD)	69.7±11.8	73.2±14.8	<0.05
Gender ratio m : f (%)	54 : 46	55 : 45	NS
BCVA (ETDRS letters±SD)	58.8±22.3	42.7±27.1	<0.001
BCVA ≤ 35 ETDRS letters *n* (%)	40 (16)	79 (32)	<0.001
CRT (μm±SD)	468±203	587±287	<0.001
Glaucoma *n* (%)	45 (18)	71 (29)	<0.05
Hypertension *n* (%)	145 (58)	163 (67)	<0.05
Diabetes mellitus *n* (%)	43 (17)	32 (13)	NS

*BCVA* best-corrected visual acuity, *ETDRS* Early Treatment Diabetic Retinopathy Study, *CRT* central retinal thickness

**Table 2:** BRVO patients: demographics and baseline ocular characteristics

Parameter	Anti-VEGF n=105	Dex implant n=35	*p* value
Age (yrs±SD)	70.3±11.0	73.4±11.8	NS
BCVA (ETDRS letters±SD)	55.7±16.2	53.6±17.4	NS
CRT (μm±SD)	547±203	531±212	NS
Pseudophakia	22(20)	10 (29)	NS
Glaucoma/OHT *n* (%)	22 (21)	0 (0)	<0.01
Hypertension *n* (%)	61 (58)	19 (54)	NS
Diabetes mellitus *n* (%)	11 (10)	9 (26)	NS

*VEGF* vascular endothelial growth factor, *Dex* dexamethsone, *BCVA* best-corrected visual acuity, *ETDRS* Early Treatment Diabetic Retinopathy Study, *CRT* central retinal thickness, *OHT* ocular hypertension

**Table 3:** CRVO patients: demographics and baseline ocular characteristics

Parameter	Anti-VEGF *n*=121	DEX implant *n*=31	*p* value
Age (yrs±SD)	74.3±11.8	72.9±15.2	NS
BCVA (ETDRS letters±SD)	41.5±22.4	56.8±13.9	<0.001
CRT (μm±SD)	696±257	615±256	NS
Pseudophakia	30(24)	11 (35)	NS
Glaucoma/OHT *n* (%)	39 (32)	4 (13)	<0.05
Hypertension *n* (%)	86 (71)	21 (68)	NS
Diabetes mellitus *n* (%)	17 (14)	6 (19)	NS

*VEGF* vascular endothelial growth factor, *DEX* dexamethsone, *BCVA* best-corrected visual acuity, *ETDRS* Early Treatment Diabetic Retinopathy Study, *CRT* central retinal thickness, *OHT* ocular hypertension.

### Time of follow-up

**Fig. 1:**
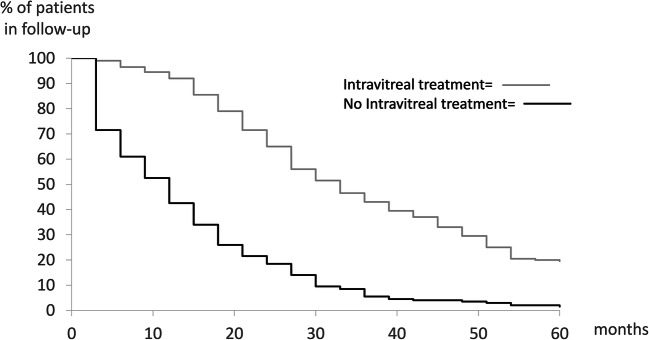
Kaplan–Meier survival curves of RVO patients still in follow-up over time. Gray graph showing patients receiving anti-VEGF therapy and black graph represent patients not receiving intravitreal treatment

**Fig. 2:**
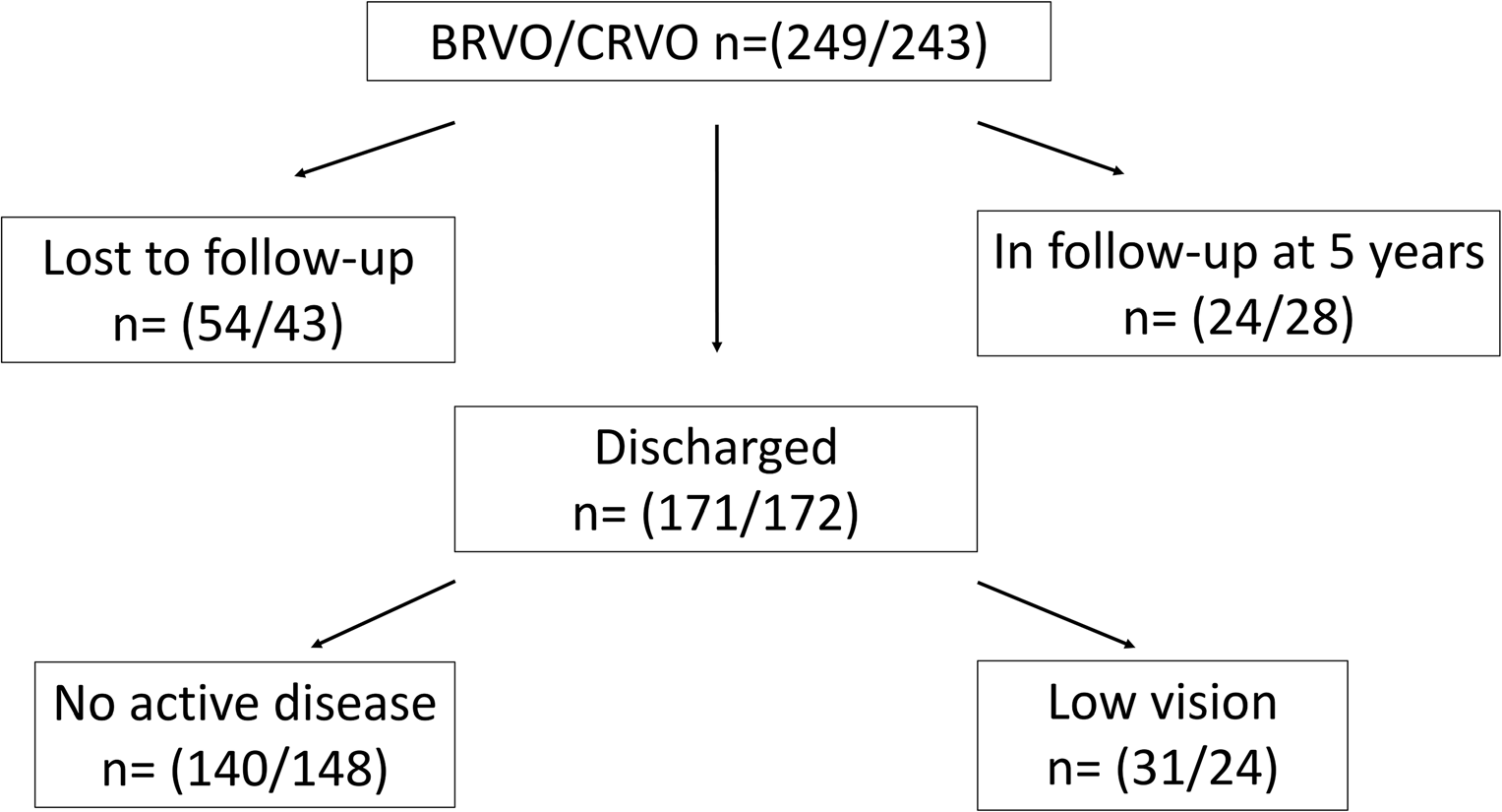
Flow chart for follow-up in RVO patients

Overall, the mean time of follow-up was 24.7±18.6 and 25.4±19.7 months in the BRVO and CRVO groups, respectively. Patients needing intravitreal injections at baseline had a mean follow-up of 33.2±17.7 months (BRVO) and 34.3±18.1 months (CRVO) compared to 13.6±13.7 months and 11.0±12.4 months in BRVO and CRVO subjects respectively not needing injections (*p*<0.0001). The percentages of patients treated for ME still in follow-up at the end of each year were 92, 62, 43, 26, and 17 for the BRVO group and 92, 68, 43, 33, and 22 for the CRVO patients (Fig. 1). After five years, 9.6% (24/249) of the BRVO patients and 12.3% (30/243) of the subjects with CRVO were still in follow-up. Different reasons for the discontinuation of the follow-up were recorded. Overall, 19.7% (97/492) of the patients were lost to follow-up. The main reason for discharge prior to the 5-year visit was lack of disease activity in 58.5% (288/492) of the subjects (Fig. 2).

### Visual acuity

**Fig. 3:**
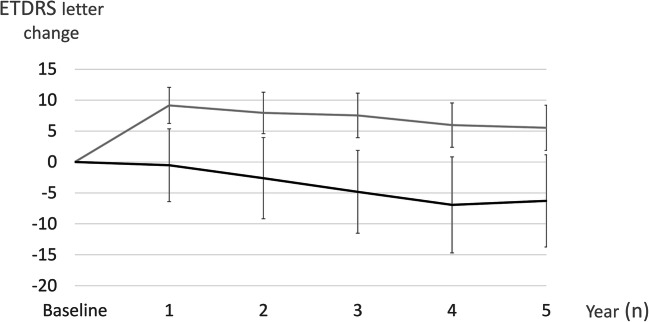
Change in visual acuity over time in all RVO patients treated with intravitreal injections. Gray graph represent patients treated with anti-VEGF injections and black graph patients receiving treatment with the DEX implant. Error bars represent 95% confidence intervals

Analyzing all patients at the end of the follow-up in the study cohort, the mean BCVA improved +4.0±16.9 letters (*p*<0.001) in the BRVO group compared to a loss of −0.7±22.1 letters (*p*=NS) in the CRVO group. A separate analysis of patients receiving intravitreal therapy showed that RVO patients treated with anti-VEGF injections improved +5.5±24.7 letters while patients receiving the DEX implant lost −6.3±28.0 letters (*p*<0.01) (Fig. 3). BRVO patients treated with anti-VEGF improved +9.8±20.4 letters while patients receiving the DEX implant lost −2.1±23.4 letters at the end of follow-up (*p*<0.05). In the CRVO group, patients treated with anti-VEGF improved +0.2±27.6 letters while patients receiving the DEX implant lost −9.7±32.6 letters (*p*=0.11). A regression analysis was calculated to predict visual outcome based on the baseline characteristics found in Table 1. We found that older age in all RVO patients (*p*<0.01) and glaucoma in BRVO patients (*p*<0.01) were significantly associated with a worse visual outcome.

### Treatment exposure

**Fig. 4:**
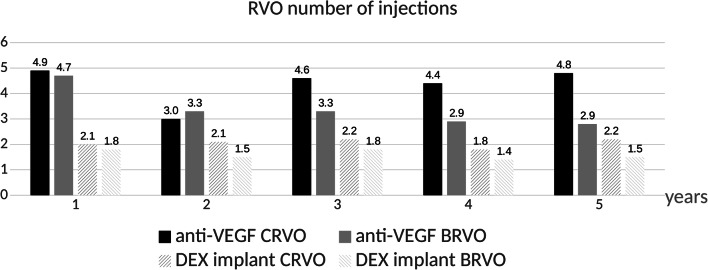
Number of injections each year in RVO patients

**Fig. 5:**
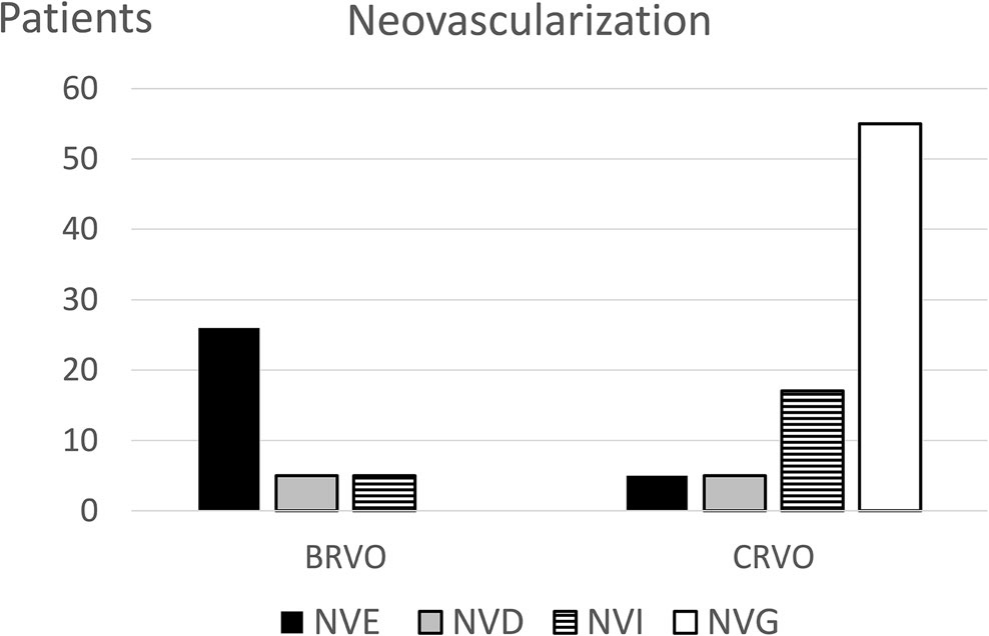
Neovascularization during follow-up in BRVO and CRVO patients. *NVE* neovascularization elsewhere, *NVD* neovascularization of disc, *NVI* neovascularization of iris, *NVG* neovascular glaucoma

Of all BRVO patients, 56% (140/249) received intravitreal therapy of which 76% (106/140) were treated with anti-VEGF injections and 24% (34/140) with a DEX implant. Each year, BRVO patients treated with anti-VEGF therapy received 4.7±1.5, 3.3±1.9, 3.3±2.0, 2.9±1.7, and 2.8±2.0 injections. Patients treated with a DEX implant received 1.8±0.8, 1.5±0.6, 1.8±0.6, 1.4±0.5, and 1.5±1.0 injections during each year (Fig. 5). Of all CRVO patients, 60% (147/243) received intravitreal therapy for ME of which 79% (115/147) were treated with anti-VEGF injections and 21% (31/140) with a DEX implant. CRVO Patients on anti-VEGF therapy received 4.9±1.6, 3.0±2.8, 4.6±2.2, 4.4±2.4, and 4.8±2.2 injections during each year. Patients treated with a DEX implant received 2.0±1.1, 2.1±0.7, 2.2±1.0, 1.8±1.0, and 2.2±0.5 injections each year (Fig. 4). In the BRVO group, 11% (12/106) of the patients were switched from anti-VEGF therapy to the DEX implant and 15% (5/34) of the subjects from the DEX implant to anti-VEGF therapy (*p*=NS). In the CRVO group, 23% (26/115) of the patients were switched from anti-VEGF therapy to the DEX implant and 19% (6/31) of the subjects from the DEX implant to anti-VEGF therapy (*p*=NS). Overall, we did not find any association between the occurrence of ME and baseline characteristics as age, glaucoma, diabetes, and hypertension.

### Anatomical outcome

Overall, in the BRVO group, the CRT decreased from 467±203 μm at baseline to 338±142 μm at the end of follow-up (*p*<0.001). In the CRVO group, the CRT decreased overall from 593±290 μm at baseline to 373±243 μm at the end of follow-up (*p*<0.001). At all time points, the CRT decreased more in the group treated with anti-VEGF injections compared to subjects receiving a DEX implant. In RVO patients treated with anti-VEGF injections, the central retinal thickness decreased to 322±174μm compared to 398±174 μm in patients treated with the DEX implant (*p*<0.05). In the BRVO group, the CRT decreased in subjects receiving anti-VEGF injections from 547±212 to 321±124 μm compared to a reduction from 531±170 to 391±177 μm in patients treated with the DEX implant (*p*<0.05). In the CRVO group, at the end of follow-up, patients receiving anti-VEGF injections, the CRT had decreased from 673±251 to 363±234 μm compared to 615±256 to 430±277 μm in patients treated with the DEX implant (*p*<0.05) (Fig. 4).

### Neovascularization

Neovascularization (NV) developed in 14% (35/249) of the BRVO patients and in 30% (74/243) of the CRVO patients. In subjects with BRVO that developed NV, neovascularization elsewhere (NVE) was the most common location seen in 71% (25/35) of patients. In subjects with CRVO that developed NV, neovascular glaucoma (NVG) was the most common presentation seen in 72% (53/74) of patients (Fig. 5).

## Discussion

Treatment with intravitreal injections does not seem to shorten the time to the ultimate resolution of ME in patients with RVO. In our study cohort, the mean time of follow-up for patients receiving intravitreal injections was 34 months. Considering that 20% of the patients in our cohort were still in follow-up after 5 years, the actual mean time to the resolution of ME is even longer. Previous natural history studies, before the intravitreal injection era, showed a mean time for ME resolution between 18 and 21 months in BRVO patients and 23 and 29 months in subjects with CRVO [2, 15]. There is some evidence that intravitreal injections can alter the natural history of RVO and prolong the time to resolution of ME. The RETAIN study that investigated the long-time outcome in RVO patients demonstrated that in order to control ME, 50% of patients with BRVO and 56% of patients with CRVO still needed ranibizumab injections 4 years after the initial treatment [9]. Several reasons may contribute to this finding. When the effect of the intravitreal injection disappears, a rebound phenomenon has been described. It is characterized by a recurrence of ME in excess of the baseline value. Studies have shown this to occur in approximately 10% of RVO patients when treated with either anti-VEGF agents or a DEX implant [16, 17]. This could contribute to a postponed resolution of ME especially in patients followed on a PRN regimen where treatment is given only when the edema recurs. Furthermore, it is well known that VEGF plays an important role in both angiogenesis and endothelial cell proliferation [18]. Green et al. showed that endothelial cell proliferation is an integral part of the process of organization and recanalization of the thrombus [19]. Hypothetically, the ultimate recovery from the RVO may take a longer time if the eye is exposed to recurrent VEGF inhibition mediated by the intravitreal injections.

Comparing the visual outcome in real-life data with prospective controlled studies is not always meaningful. The patient population in trials with strict inclusion and exclusion criteria and a stringent follow-up regimen can be fundamentally different from a real-life cohort. Real-life data tend to include a patient population that is far more representative of unselected patients than randomized controlled trials [20, 21].

In our cohort including 221 patients treated with anti-VEGF injections, we found that given over five years on a PRN dosing regimen a substantial and continuous visual benefit in patients with BRVO. Patients gained a mean of +9.8 letters at the end of follow-up. Subjects with ME secondary to CRVO had a significantly worse outcome improving +0.2 letters after five years. Other real-life studies report similar results. The BERVOLT study that retrospectively followed 152 patients treated with bevacizumab for a mean of 24–26 months found that patients with BRVO improved +12.5 letters (0.25 Log MAR units) while patients with CRVO lost −5.9 letters (−0.118 Log MAR units) [11, 22] . Chatziralli et al. investigated retrospectively the outcome in 54 RVO patients treated with ranibizumab. After a mean follow-up of 47 months, patients improved +15.1 letters (BRVO) and +6.9 letters (CRVO) [10]. In a large retrospective analysis of 351 eyes with RVO treated with anti-VEGF injections for up to five years, a small mean loss of visual acuity was observed at the end of follow-up. Unfortunately, the cohort was not sub-divided into BRVO and CRVO patients [23]. In common for all these real-life studies is that patients with BRVO improved approximately two ETDRS lines more than subjects with CRVO.

Overall, we found a significantly better visual and anatomical outcome in patients treated with anti-VEGF agents compared to a DEX implant. Both BRVO and CRVO patients treated with anti-VEGF agents had a significant, almost two-line better visual outcome compared to subjects treated with a DEX implant. Similar results have been found in several studies. The COMRADE trials with a one-year follow-up compared treatment with ranibizumab and a DEX implant. Patients treated with ranibizumab improved +9.9 and +5.4 letters more (BRVO and CRVO respectively) than subjects receiving a DEX implant [24]. Comparable results were found in the COMO study that prospectively followed BRVO patients for 12 months. Patients treated with ranibizumab gained +10.0 letters more than subjects receiving a DEX implant did [25].

The difference in the visual outcome between patients treated with anti-VEGF agents and the DEX implant can be due to several reasons. In our study, most eyes treated with the DEX implant were phakic probably causing a cataract-associated attenuation of BCVA improvement. Furthermore, the patients treated with the DEX implant had a less favorable anatomical outcome at each time point during the follow-up. Previous studies have shown greater fluctuation of mean CRT in DEX-treated patients, which can explain the worse anatomical outcome when the patients were evaluated at pre-specified time points [23–26]. The fluctuations in the CRT may be caused by too few injections of the DEX implant. In our study cohort the patients received a mean of 1.6 (BRVO) and 2.1 (CRVO) yearly DEX injections. In clinical practice, DEX re-implantation is required earlier than six months in many patients. Bezatis et al. showed that retreatment after 16 weeks was necessary in 50% of patients with RVO in order to treat the recurring ME [27]. However, it is unclear how a more aggressive treatment approach in our cohort with the DEX implant would influence the visual outcome.

Interestingly, in our study cohort, the incidence of BRVO and CRVO was similar. Previous large population-based studies have shown that the incidence of BRVO is around 3–4 times higher than CRVO [28, 29]. This discrepancy is probably due to a substantial number of asymptomatic individuals not seeking medical evaluation or patients with non-macular involvement not needing referral for intravitreal treatment. In the Stockholm region, basically all intravitreal therapy is done at two hospitals, of which Sankt Erik Eye Hospital is responsible for two thirds. RVO patients not needing intravitreal treatment may continue their follow-up with an ophthalmologist in the community.

There are several limitations with our study. This was a retrospective study without a strict follow-up protocol. A substantial part of the patients was lost to follow-up but over 80% remained in the study and were followed for up to five years. Retreatment was decided based on change in BCVA and ME but treatment decisions were at the physician’s discretion. However, this is the reality of common practice and we believe that real-life data can provide important information to the physician, patients, and health-care decision makers concerning prognosis, long-term efficacy, and economic assessment.

In conclusion, we found that almost half of the patients receiving intravitreal treatment for ME secondary to RVO were still in treatment after 3 years. BRVO patients treated with intravitreal injections improved significantly more than subjects with CRVO. Patients receiving anti-VEGF therapy had a significantly better visual and anatomical outcome compared to patients treated with a DEX implant.
